# Convergent approaches toward the study of multisensory perception

**DOI:** 10.3389/fnsys.2013.00081

**Published:** 2013-11-08

**Authors:** Diana K. Sarko, Dipanwita Ghose, Mark T. Wallace

**Affiliations:** ^1^Department of Anatomy, Cell Biology and Physiology, Edward Via College of Osteopathic MedicineSpartanburg, SC, USA; ^2^Department of Anesthesiology, Vanderbilt University Medical CenterNashville, TN, USA; ^3^Department of Hearing and Speech Sciences, Vanderbilt UniversityNashville, TN, USA

**Keywords:** electrophysiology, multisensory, oscillations, receiver operating characteristics, spike synchrony

## Abstract

Classical analytical approaches for examining multisensory processing in individual neurons have relied heavily on changes in mean firing rate to assess the presence and magnitude of multisensory interaction. However, neurophysiological studies within individual sensory systems have illustrated that important sensory and perceptual information is encoded in forms that go beyond these traditional spike-based measures. Here we review analytical tools as they are used within individual sensory systems (auditory, somatosensory, and visual) to advance our understanding of how sensory cues are effectively integrated across modalities (e.g., audiovisual cues facilitating speech processing). Specifically, we discuss how methods used to assess response variability (Fano factor, or FF), local field potentials (LFPs), current source density (CSD), oscillatory coherence, spike synchrony, and receiver operating characteristics (ROC) represent particularly promising tools for understanding the neural encoding of multisensory stimulus features. The utility of each approach and how it might optimally be applied toward understanding multisensory processing is placed within the context of exciting new data that is just beginning to be generated. Finally, we address how underlying encoding mechanisms might shape—and be tested alongside with—the known behavioral and perceptual benefits that accompany multisensory processing.

## Introduction

Because we live in a world composed of a complex amalgam of sensory information, it is only through the ability to combine the various forms of this information that a meaningful behavioral and perceptual gestalt (an organized “whole” greater than the sum of its parts) can be formed. Furthermore, the adaptive advantages that multisensory integration confers are critical to survival, and often allow appropriate behavioral responses to be generated under circumstances in which information from one sense is inadequate. Enhanced orientation (Stein et al., 1988, 1989), improved target detection (Frassinetti et al., 2002; Lovelace et al., 2003), and faster responses (Hershenson, 1962; Hughes et al., 1994; Frens et al., 1995; Harrington and Peck, 1998; Murray et al., 2001; Corneil et al., 2002; Forster et al., 2002; Molholm et al., 2002; Amlot et al., 2003; Diederich et al., 2003) are among the multitude of behavioral benefits seen when information is combined from two or more sensory modalities.

These behavioral and perceptual changes invoked under multisensory conditions reflect a series of neural computations involving the convergence and integration of inputs from the different sensory modalities. The presence of such convergence and integration can be inferred from the fact that behavioral responses are often faster than those predicted by a simple probability summation of the responses to the sensory cues presented individually (Hughes et al., 1994, 1998; Corneil and Munoz, 1996; Harrington and Peck, 1998). Further evidence for links between neural activity and its behavioral correlates have been seen when comparing and relating the activity of multisensory neurons to behavioral responses. For example, stimulus combinations that enhance the activity of multisensory neurons in the superior colliculus (SC) also enhance an animal's orientation abilities (Stein et al., 1988, 1989). Spatially and temporally coincident audiovisual stimulus combinations improve the animal's ability to detect and approach the correct location, whereas spatially disparate stimuli reduce the percentage of correct responses (Stein et al., 1988, 1989). Furthermore, when stimulus intensity is manipulated, the least effective stimuli (e.g., a dim LED that yields a low neuronal response) produce the greatest behavioral gains (Stein et al., 1989).

## Multisensory processing: classical neurophysiological analyses

Although there is a high degree of multisensory convergence at many sites throughout the central nervous system, the foundation for examining the physiological underpinnings of multisensory integration has focused on a midbrain structure, the SC (e.g., Stein and Meredith, 1993). The reasons for the choice and preeminence of this model are manifold, but include its high incidence of multisensory neurons, well-established topographic organization, and well-characterized role in mediating orientation movements of the eyes and head. Using the SC as a model, Stein and Meredith conducted seminal studies that characterized the basic principles by which multisensory neurons synthesize their inputs from multiple modalities (Meredith and Stein, 1983, 1985, 1986a,b, 1996; Meredith et al., 1987; Stein, 1988; Stein et al., 1988). Their work showed that multisensory (e.g., visual-auditory) stimulus pairs presented in close spatial and temporal proximity typically resulted in large response enhancements, a gain that makes intuitive sense given that stimuli resulting from a singular event are bound by common spatial and temporal properties (Meredith and Stein, 1983, 1986a; Meredith et al., 1987; Wallace et al., 1997, 1998; Jiang et al., 2002; Burnett et al., 2004). Furthermore, they showed that the largest response enhancements were seen with the pairing of weakly effective stimuli, whereas combining increasingly effective stimuli yielded less gain (Perrault et al., 2005; Stanford et al., 2005). This principle, known as *inverse effectiveness*, also makes intuitive and ethological sense given that response amplification from the additional sensory signal becomes unnecessary when one signal alone is highly salient, and therefore sufficient. Whereas such midbrain studies targeted the deep layers of the SC—where inputs from auditory, somatosensory, and visual modalities converge—recent studies have assessed more perceptual aspects of multisensory processing by targeting cortical areas such as the anterior ectosylvian sulcus (AES) of cats and ferrets, or the posterior parietal cortex, particularly the superior temporal sulcus (STS) of primates (for review, see Stein and Stanford, 2008). In contrast to mediation of head and eye movements directly involved with the SC, cortical multisensory areas are guided by similar substrates (changes in activity related to spatial and temporal congruence as well as stimulus efficacy) to mediate *perceptual* binding, including cross-modal cues involving congruent motion and audiovisual vocal communication (Barraclough et al., 2005; Royal et al., 2009).

The focus of these early neurophysiological response analyses of multisensory neurons [defined as neurons that respond to, or are influenced by, stimuli from more than one sensory modality (Stein and Stanford, 2008)] was on the mean number of spikes evoked per stimulus presentation (analyzed as single unit activity, or SUA). Using spike count metrics, this work characterized the responses to both single modality (i.e., visual alone, auditory alone) and combined modality (i.e., visual-auditory) stimulation, and used this to gauge whether multisensory stimuli resulted in *response enhancement* (a significant increase in the mean number of spikes when compared with the most effective unisensory stimulus), *response depression* (a significant decrease relative to the best modality response), or no interaction between the sensory modalities (Meredith and Stein, 1983, 1986b; Meredith et al., 1987; Wallace et al., 1996, 1998; Jiang et al., 2002; Burnett et al., 2004). To quantify the magnitude of these effects, the *interactive index* (% interaction, or ii) (Meredith and Stein, 1983) is calculated as:

$$[(CM-SM_{\text{max}})/SM_{\text{max}}]\times 100=\%\text{ interaction}$$

where *CM* is the mean number of spikes per trial evoked by combined-modality stimulation and *SM*_max_ is the mean number of spikes evoked by the most effective single-modality stimulus. The power of the interactive index is that it shows the gain (or loss) of response attributable to the presence of a stimulus in a second sensory modality—thereby demonstrating the presence or absence of a *multisensory interaction*—a measure with undeniable ethological validity.

## Multisensory processing: additional analytical approaches

One shortfall of the interactive index measure is its use of the strongest unisensory response alone as the comparator, which fails to incorporate the influence of the second sensory modality. To circumvent this concern, analyses structured around an additive model began to be commonly used. This method creates a predicted multisensory response based on the addition of the two unisensory responses, which can then be contrasted against the actual observed response using the mean statistical contrast (or *multisensory contrast*, msc) measure:

$$\sum \text{[}(SA-SM_{1})-(SM_{2}-CM)]/n=\text{mean statistical contrast}$$

where *SA* is the spontaneous activity, *SM*_1_ is the first single-modality response (e.g., auditory), *SM*_2_ is the second single-modality response (e.g., visual), *CM* is the combined-modality response, and *n* is the number of trials. In each case the response is defined as the mean number of spikes per trial evoked for the duration specific to each response (*SM*_1_, *SM*_2_, and *CM*, generally using 10 ms bins) while *SA* is the average spikes per trial during 500 ms interval prior to stimulus onset. Using this equation, only responses that exceed the level of spontaneous activity affect the mean statistical contrast. This model assumes independence between inputs from each sensory modality and utilizes additive factors logic to distinguish between *superadditive* (contrast > 0) and *subadditive* (contrast < 0) responses (Perrault et al., 2003, 2005; Stanford et al., 2005). Thus, msc characterizes the type of integration present, beyond simply determining enhancement vs. depression of the response, by incorporating both component unisensory responses—rather than only the strongest—as a metric for classifying integration effects.

Although mean statistical contrast is a powerful tool to measure multisensory integration (and can be complemented with use of the interactive index), it must be recognized that these metrics still rely on changes in the mean firing profile of the neurons under study. Studies within sensory systems have illustrated that information can be encoded in forms that differ from these traditional spike-based measures—a series of findings that are beginning to be extended into multisensory systems. Alternative measures such as mean response duration, response latency (measured either as mean response latency or first spike latency), and peak firing rate (measured from the time bin in which the maximum number of spikes occurred post-stimulus) are also used to quantify multisensory integration. These measures provide valuable insights into temporal response dynamics and their effect on the integrative capacity of multisensory neurons (Meredith et al., 1987; Royal et al., 2009; Ghose et al., 2012). Together with measures of response variability (further described below), such metrics help to reveal encoding strategies that may not be evident in studying firing rate changes alone.

## Changes in neuronal response variability: Fano factor analysis

An integral concept when studying sensory (and multisensory) systems is that of reliability. In psychophysical studies, reliability is generally framed from the perspective of cue weighting, with the relative cue weights being a function of the reliability of the various sensory inputs (Ernst and Banks, 2002; Shams et al., 2005; Burr and Alais, 2006; Burge et al., 2010). Observers of sensory stimuli tend to employ an optimal strategy that weights each cue in proportion to its reliability, a behavioral finding supported by predictive neural population responses as well (Fetsch et al., 2012). Cue reliability also has strong relevance for multisensory encoding. In a simple multisensory context, one can envision a situation in which one of the sensory inputs (e.g., vision) is providing much more reliable information than the other modality (e.g., audition), and hence should be weighted more in an evaluation of the sensory evidence, such as during the localization of an object in space. The concept of cue reliability can be readily extended into the neural domain, in which the metric of interest is the variability of the neuronal response (in essence the opposite of reliability, since the variability of the response would directly impact how reliably a stimulus is encoded) upon repeated stimulus presentations. Response variability of spike counts is captured in the *Fano factor* (FF) calculation:

$$FF=\sigma^{2}/\mu$$

in which the ratio of variance (σ^2^) to the mean (μ) of spike counts is computed across trials and averaged over a specific time window of single unit neuronal response (Fano, 1947). A *FF*-value of one indicates neuronal responses that are as reliable as would be found from a Poisson process (Fano, 1947; Softky and Koch, 1993; De Ruyter Van Steveninck et al., 1997; Kara et al., 2000; Eden and Kramer, 2010). It is important to note that the level of neuronal activity (magnitude of firing rate) plays an important role in the determination of FF, since mean firing rate is incorporated into the FF calculation. Thus, at high firing rates, responses are typically less variable (Tolhurst et al., 1983; Softky and Koch, 1993; Holt et al., 1996; Kara et al., 2000; Carandini, 2004; Gur and Snodderly, 2006). One reason for this decline in variability at very high firing rates is the refractory period, which constrains the temporal profile of a response (Berry and Meister, 1998; Kara et al., 2000). Recent studies have also shown that *FF*-values can vary depending on the brain region and the effectiveness of the sensory stimulus (Kara et al., 2000; Gur and Snodderly, 2006; Kayser et al., 2010; Mochol et al., 2010). For instance, in the cat SC, FF has been used to distinguish between parallel processing channels (W and Y, with superficial layer neurons receiving inputs from each channel) such that slow-moving visual stimuli showed increased *FF*-values with increased firing rate, whereas fast-moving stimuli resulted in *FF*-values that correlated negatively with firing rate (Mochol et al., 2010).

**Figure 1:**
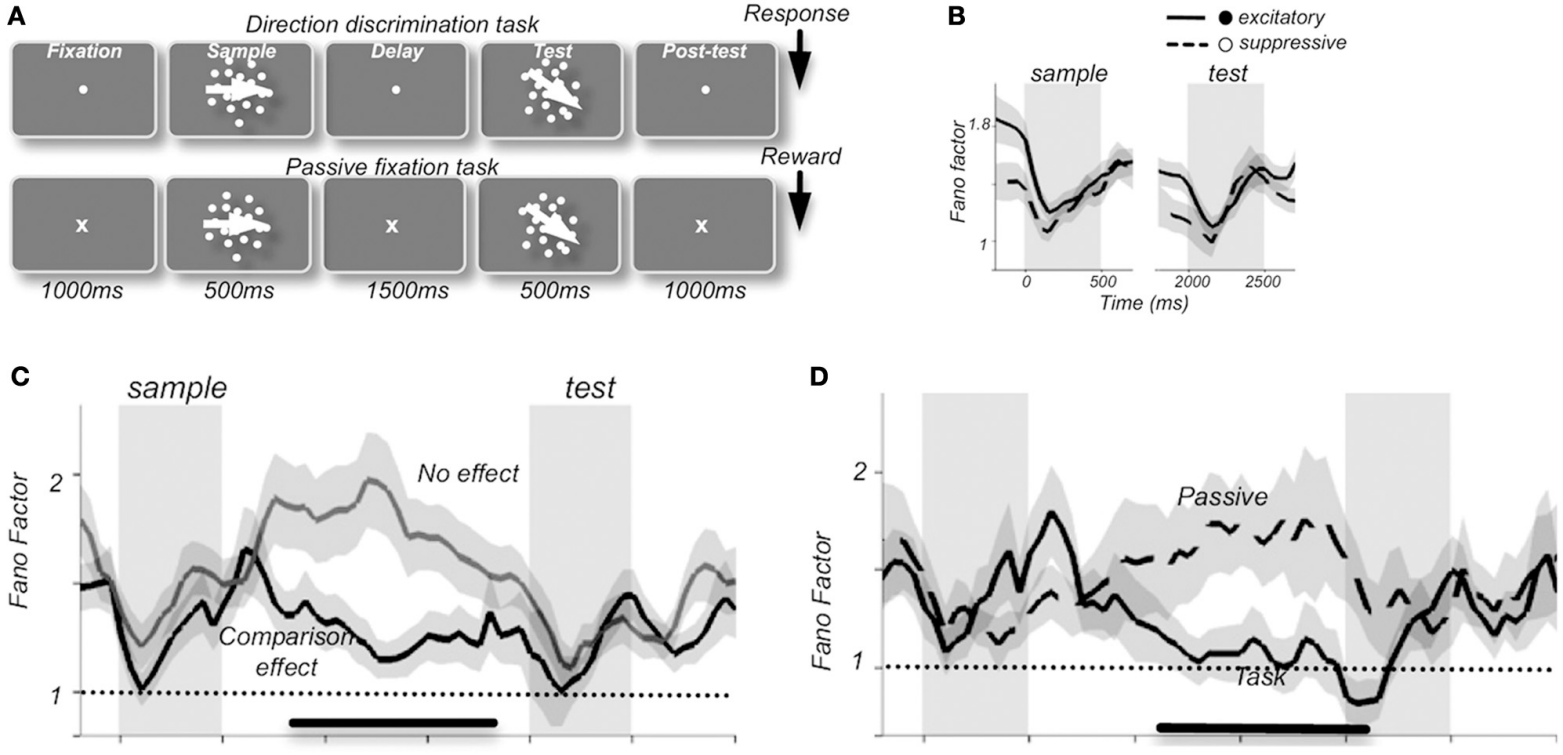
**Average Fano factor values change with the components of a motion discrimination task (A) in neuronal recordings from monkey prefrontal cortex**. A stimulus-induced drop in FF was present compared to fixation, delay, and post-test periods **(B)**, along with a preparatory drop in FF preceding the test component in neurons that were able to discriminate between sample and test stimuli **(C)**. Fano factor values also varied with behavioral performance such that higher variability was observed during passive fixation conditions than during the visual discrimination task, an effect that was thought to reflect the level of engagement in the task **(D)**. “Excitatory” and “suppressive” **(B)** refers to 2 classes of neurons: excitatory broad-spiking putative pyramidal cells with spike durations of >200 μs and narrow-spiking putative inhibitory interneurons with spike durations of <200 μs, respectively [reprinted with permission from Hussar and Pasternak (2010)].

Changes in response reliability are potentially very meaningful from an information encoding perspective, as they could be used as weighting factors in neural processes responsible for cue combination (Fetsch et al., 2012). One tangible example of the use of FF as a tool has come from studies that have tied response variability to functional relevance and behavioral outcomes. In the prefrontal cortex of macaques, *FF*-values were shown to change during the components of a motion discrimination task (Figure 1). Thus, a stimulus-induced drop in FF was present when compared to fixation, delay, and post-test periods, along with a preparatory drop in FF preceding the test component in neurons that were able to discriminate between sample and test stimuli (random-dot stimuli, with the “sample” phase intended for identification and memorization of stimulus direction and the “test” phase intended for comparison in order to assess accuracy of direction discrimination by varying the difference between directions in each). *FF*-values also varied with behavioral performance such that higher variability was observed during passive fixation conditions compared to visual discrimination tasks, an effect that is thought to reflect the level of engagement in the task (Hussar and Pasternak, 2010).

**Figure 2:**
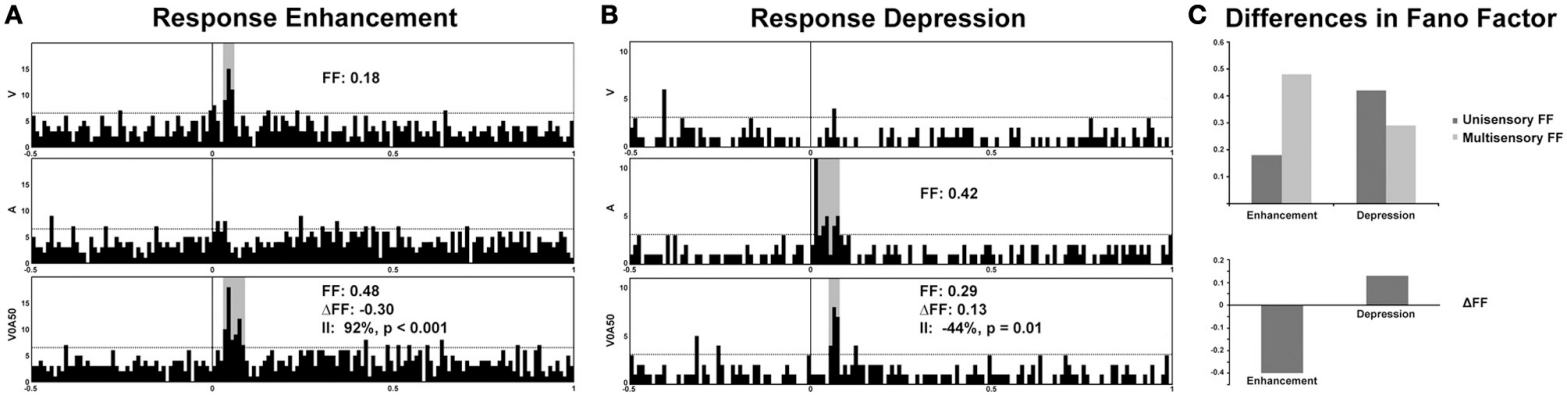
**Response variability in representative multisensory neurons from electrophysiological recordings in the superior colliculus of an awake cat**. Note the significant enhancement of response under multisensory conditions (visual-auditory) compared to the best unisensory condition (92% enhancement of firing rate denoted by the Interactive Index, II; **(A)** Preliminary evidence from our laboratory demonstrates an increase in response variability (Fano factor, or FF) in multisensory conditions exhibiting response enhancements, whereas a decrease in variability (increased reliability) is observed for response depressions **(B,C)**. A, auditory, V, visual, and V0A50 denotes audiovisual stimuli presented with a temporal offset of 50 ms.

Recent studies from our laboratory have begun to highlight the utility of FF analysis as a tool for elucidating information content in multisensory systems. In recordings from the SC of both awake and anesthetized cats, different modes of multisensory interactions (i.e., enhancement vs. depression) were discovered to be associated with distinctly different changes in FF. Thus, whereas response enhancements are accompanied by an increase in response variability under multisensory conditions, response depressions are characterized by decreased variability (Figure 2) (Sarko et al., 2012). We assess this by calculating the change in *FF*-values (Δ*FF*) between the maximum unisensory response (*U*_*ff*_, unisensory *FF*-value) and the multisensory response (*M*_*ff*_, multisensory *FF*-value):

$$\Delta FF=U_{ff}-M_{ff}$$

whereas a positive Δ*FF*-value indicates unisensory responses that are more variable (less reliable) than multisensory responses, a negative Δ*FF*-value indicates the reverse. On average, neuronal responses that demonstrated response enhancements under multisensory conditions were less reliable (with higher *FF*-values) compared to unisensory responses, resulting in negative Δ*FF*-values (Figures 2A,C). In contrast, response depressions were associated with positive Δ*FF*-values indicative of greater response reliability and lower *FF*-values under multisensory conditions (Figures 2B,C). This suggests that response variability offers an additional neural encoding mechanism beyond firing rate alone, and runs somewhat counter to what would be predicted based on changes in firing rate alone—that responses should be more reliable under multisensory enhancement conditions. It may be that high firing rates are sufficient to bind multisensory stimuli under conditions of response enhancement, whereas more reliably encoded multisensory stimuli are necessary in conditions that produce weak multisensory responses. It has further been shown that encoding variability may differ according to behavioral states of discrimination vs. detection, with detection possible despite high response variability and discrimination more reliant on spike timing precision (Reich et al., 1997). Thus, by extension, because multisensory response enhancements are associated with greater neuronal response variability, they may be more behaviorally relevant for accurate detection of multisensory stimuli. Furthermore, response depressions (associated with decreased variability, or greater reliability) may be more relevant in discrimination of multisensory stimuli (e.g., discriminating the spatially disparate stimuli that are known to elicit response depressions). This could be behaviorally tested by combining neural recordings in the AES with a saccade task in which the animal is presented with audiovisual stimuli that are moving either congruently (in the same direction) or incongruently under varying degrees of motion coherence, thereby titrating cue reliability. One prediction from such experiments would be that the activity of multisensory neurons would reflect behavioral outcomes (faster reaction times and enhanced accuracy of saccade direction matched to the most reliable cue condition). Neurons tuned to a particular direction—for instance, left—would exhibit greater firing rates when the more reliable cue was moving left, and greater multisensory enhancement when both cues were moving congruently. In contrast the introduction of less motion coherence would likely result in greater neuronal response variability, slower reaction times, and impaired accuracy in saccade direction.

Recent studies by Kayser et al. have further illustrated the importance of response variability as a possible information source under multisensory circumstances. In recordings from primate auditory cortex, they showed that naturalistic audiovisual stimuli and their degree of congruence play an important role in response variability and information gain (Kayser et al., 2010). Epochs of weak auditory response became more variable with the addition of visual input, whereas epochs of strong auditory response became less variable and more reliable under multisensory conditions. This in turn had a direct effect on the information encoded, with information gain directly related to increased response reliability. This information gain decreased when auditory and visual cues were mismatched, reflecting the dependence on feature matching between modalities rather than the addition of a visual stimulus alone, and linking analyses of response variability and information gain to perceptual meaning.

## Changes in synaptic processes: local field potentials

Although the emphasis of multisensory work has been on neurons that are overtly responsive to stimuli from two or more sensory modalities, recent studies have highlighted that multisensory interactions can manifest in neurons that are only overtly responsive to one sensory modality, with the second *modulating* responses of the driving modality (Schroeder et al., 2001, 2003; Schroeder and Foxe, 2002; Foxe and Schroeder, 2005; Ghazanfar et al., 2005; Carriere et al., 2007, 2008; Allman et al., 2008; Krueger et al., 2009; Meredith and Allman, 2009). Indeed, a recent paradigm shift in the multisensory field has stemmed from the suggestion that such modulatory influences can impact sensory processes even in very early sensory cortical domains typically characterized as unisensory (Morrell, 1972; Giard and Peronnet, 1999; Foxe et al., 2000; Schroeder et al., 2001; Falchier et al., 2002; Molholm et al., 2002; Schroeder and Foxe, 2002; Fu et al., 2003; Rockland and Ojima, 2003; Besle et al., 2004; Brosch et al., 2005; Ghazanfar et al., 2005; Ghazanfar and Schroeder, 2006; Kayser and Logothetis, 2007).

**Figure 3:**
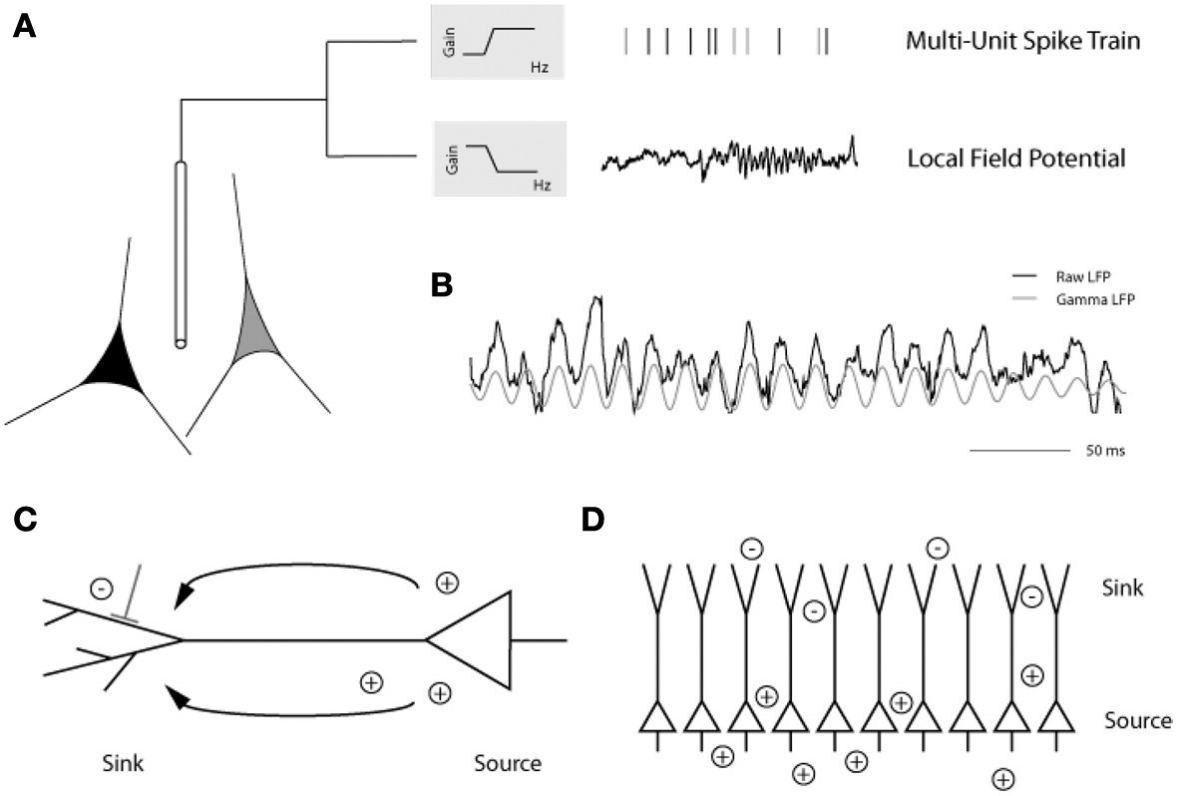
**(A)** Placing an extracellular electrode in the brain measures the mean extracellular field potential originating from the activity of a population of neurons surrounding the electrode tip. The signal is high-pass filtered and the resulting multiunit action potentials are detected. The local field potential (LFP) is the low frequency component (up to 200 Hz) of the signal with a frequency composition that varies over time. Here, prominent gamma band oscillations (between 30 and 90 Hz) are visible in the later part of the LFP trace. **(B)** In this example from the primary visual cortex of an awake monkey, the raw trace (black) has been filtered to isolate the gamma LFP (gray) prominent during visual stimulation. **(C)** Depiction of a pyramidal cell showing the current sink at the dendritic tree and the current source at the soma. **(D)** Alignment of pyramidal cells creating an open field arrangement such that synchronized synaptic input can produce strong dipoles without current flowing from individual cells canceling each other [figure reprinted with permission from Berens et al. (2010)].

Analysis of local field potentials (LFPs) elucidates subthreshold influences through sampling pooled voltage changes (Berens et al., 2008a,b, 2010). The low frequency component of the LFP signal (<200 Hz) has been the focus of the majority of LFP experiments and is believed to reflect excitatory and inhibitory postsynaptic potential changes, in addition to subthreshold membrane oscillations and after-potentials of somatodendritic action potentials, in the vicinity of the electrode tip (Mitzdorf, 1985, 1987; Kamondi et al., 1998; Buzsaki et al., 2002; Logothetis, 2003, 2008; Hasenstaub et al., 2005; Berens et al., 2008b; Trevelyan, 2009) (but see also Kajikawa and Schroeder, 2011, regarding the sampling area of LFPs). Standard extracellular recording methods can be used to detect both spiking activity and the LFP—depending on the filtering parameters applied to the signal—with each carrying distinct functional implications (Figure 3). In addition to its amplitude and latency, the raw LFP signal can be decomposed by Fourier analysis into its component frequency bands (*delta*, 1–4 Hz; *theta*, 4–8 Hz; *alpha*, 8–12 Hz; *beta*, 12–30 Hz; and *gamma*, >30 Hz) similar to those characteristic of electroencephalogram (EEG) studies. Spectral analysis of the LFP signal further assesses changes in the power spectrum of particular frequency bands that coincide with distinct stimulus conditions (Henrie and Shapley, 2005).

## Local field potentials and current source density examination of unisensory processing

Much that is known about the role of LFP modulations in sensory processing comes from work restricted to the individual sensory modalities. The onset of visual stimulation causes a shift from low-frequency to fast gamma LFP oscillations in the primary visual cortex (V1) of awake macaques (Berens et al., 2008a,b). In primate V1, gamma band activity shows the highest stimulus selectivity associated with coding stimulus features such as orientation preference and ocular dominance (Berens et al., 2008a,b). Moreover, gamma power has been reported to increase in different visual areas during perceptual (Gail et al., 2004; Wilke et al., 2006), memory-associated (Pesaran et al., 2002), and attentional (Fries et al., 2001; Taylor et al., 2005; Fries et al., 2008) processes. Feature selectivity (for both stimulus direction and speed) (Liu and Newsome, 2006), attentional allocation (Fries et al., 2001; Taylor et al., 2005), and object category selectivity (Kreiman et al., 2006) have also been related to changes in LFPs in visual cortex. Furthermore, attention to a visual stimulus results in enhanced oscillatory coupling at gamma frequencies in the frontal eye field (FEF) and area V4, which in turn is thought to optimize the postsynaptic impact of spikes from one area to the other and improve communication between the areas during attention (Gregoriou et al., 2009). In the auditory system, primary auditory cortex (A1) of awake rhesus monkeys demonstrates frequency tuning profiles that are matched across high frequency LFP and single or multi-unit activity domains (Kayser et al., 2007). In the somatosensory system, high gamma LFP oscillations are closely synchronized with the occurrence of action potentials in SII of awake monkeys, suggesting that high gamma power in LFPs may be an index of population firing rate (Ray et al., 2008a,b). Because LFPs are essentially an index of local synaptic processing, they provide information about local inputs to a given brain area (Pesaran, 2009), thereby creating an essential bridge between analyzing inputs to and outputs from a region of interest by linking LFP and spiking activity. Such studies conducted in the principal sensory modalities have established important relationships between firing rate and LFP encoding of stimulus properties, as well as perceptual and attentional correlates of LFP activity that can be extended to multisensory applications.

Although LFP fluctuations provide an important window into synaptic function that complements spiking information, there are conceptual caveats that must be considered when interpreting LFP signals. One of the most important of these is the high degree of lateral (~200–400 μm) and vertical (several mm) spread of the LFP (Kajikawa and Schroeder, 2011), which is substantially greater than had been originally estimated (Katzner et al., 2009; Xing et al., 2009), and which is undoubtedly due to volume conduction (Mitzdorf, 1985; Nunez et al., 1991; Schroeder et al., 1995; Kocsis et al., 1999). This complicates the spatial interpretation of LFP recordings in attempting to localize activity to specific regional confines as an index of underlying synaptic processes, since the observed LFP activity reflects a mixture of both local and relatively distant electrophysiological events. A recent study utilizing a detailed biophysical modeling approach has investigated the spatial extent of LFP signal spread and suggests that it depends on a variety of factors including neuronal morphology, synapse distribution, and synaptic activity correlation (e.g., uncorrelated synaptic activity produces less spatial spread than correlated activity) (Linden et al., 2011). Multicontact electrodes can be used to circumvent this issue by measuring LFPs at a variety of depths for a single penetration, spanning the cortical thickness of a given region and allowing a laminar analysis of the LFP. In particular, the spatial derivative of these LFP signals can be used to create a current source density (CSD) profile, revealing current “sources” and “sinks” (for calculations, see Nicholson and Freeman, 1975; Tenke et al., 1993). The CSD also reflects subthreshold synaptic currents but avoids the spatial confounds associated with the LFP, and also has been shown to have stimulus selectivity comparable to that of multiunits [e.g., narrow response tuning to best frequency—comparable to that of multiunit activity—during recordings from A1 of macaques, in contrast to LFP signals that demonstrated wider tuning (Kajikawa and Schroeder, 2011)]. Perhaps most importantly, CSD analysis allows a view the laminar flow of information within a specific cortical circuit by attenuating far-field contamination (i.e., volume conduction confounds).

## Local field potentials: implications for multisensory processing

**Figure 4:**
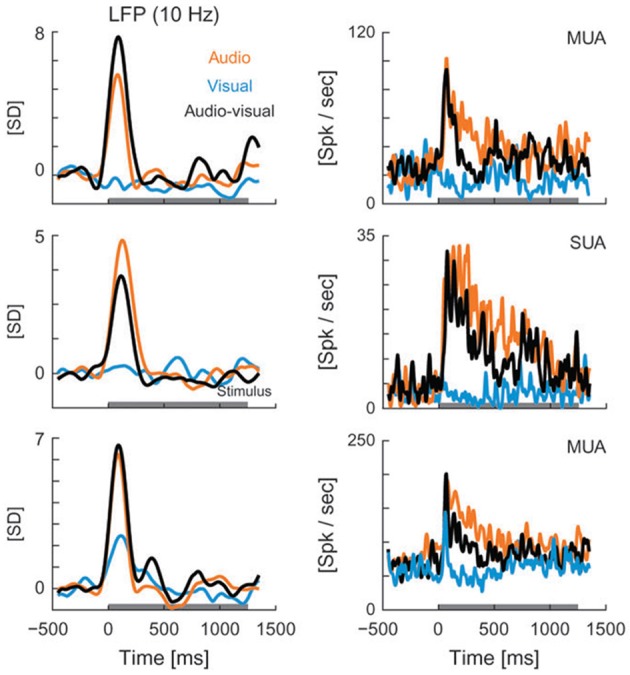
**LFPs (left column) and spiking activity (right column) recorded from auditory cortex in rhesus monkeys presented with visual (blue), auditory (orange), and audiovisual (black) stimuli**. LFP curves demonstrate the mean values for each of the 3 stimulus conditions, with the horizontal gray line along the x axis representing the stimulus interval. LFP values are shown in units of standard deviation (SD) from baseline (z-score). MUA, multi-unit activity; Spk, spikes; SUA, single-unit activity [reprinted with permission from Kayser et al. (2008)].

**Figure 5:**
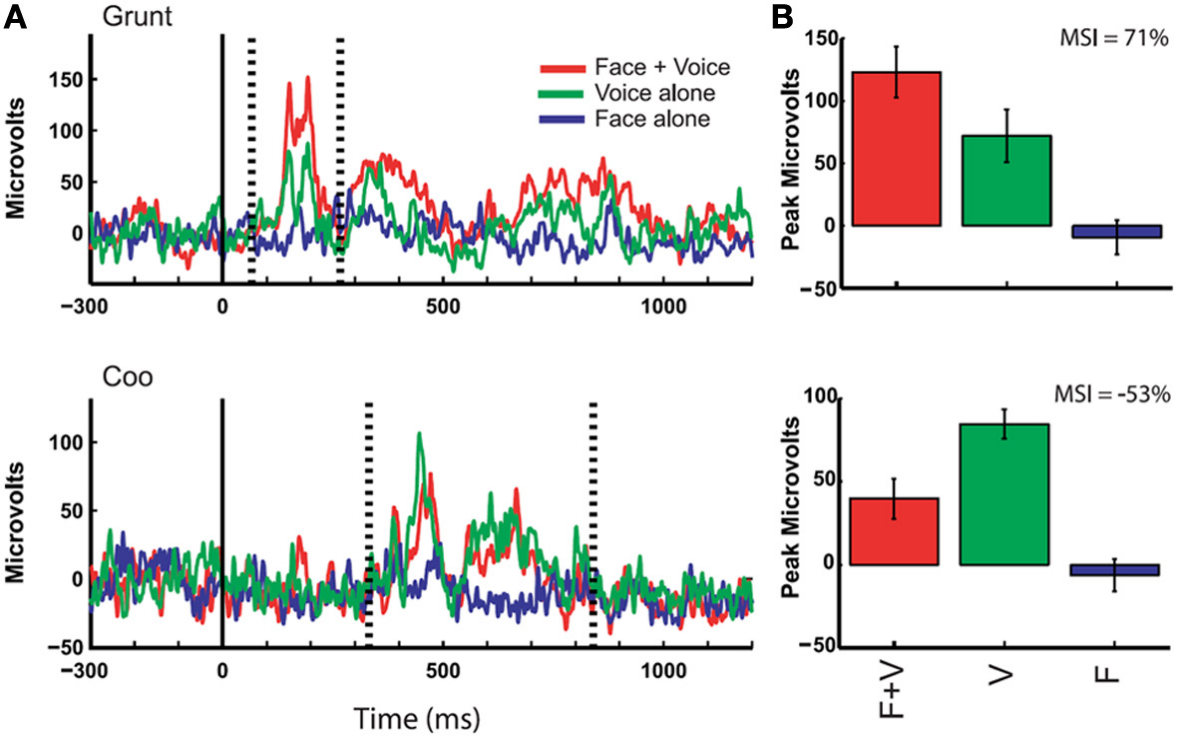
**Responses of auditory cortex (core region) to multimodal vocalizations**. Rectified local field potentials compare responses to face + voice (F + V), voice alone (V), and face alone (F) components of coos and grunts (non-human primate vocalizations commonly emitted during affiliative social interactions) in order to examine multisensory integration of social signals through communication. LFPs were recorded in auditory cortex of awake behaving rhesus monkeys while they viewed vocalizing conspecifics, and integration of faces and voices was observed through changes in LFP activity. **(A)** The solid vertical line indicates the onset of the face stimulus whereas the dotted vertical lines indicate the onset and offset of the voice stimulus (mean across 10 trials with baseline activity subtracted). Bar graphs show mean and SEM of the maximum response (F + V or V alone using a 20 ms window) between voice onset and offset compared to other stimulus conditions. **(B)** Multisensory integration index (MSI) was computed for each example and demonstrates a response enhancement (top) vs. suppression (bottom) [reprinted with permission from Ghazanfar et al. (2005), Figure 2, p 5007].

Though LFP analysis has been widely used within individual sensory systems, its application to the multisensory realm remains limited. Recent studies have begun to examine changes in the amplitude and frequency of LFP oscillations in different brain areas in response to multisensory stimuli. For instance, Kayser et al. (2008) reported visual modulation of activity in the auditory cortex of rhesus monkeys, demonstrated by changes in the amplitude of the LFP signal under cross-modal conditions. The authors demonstrate that visual stimuli modulate auditory processing in both primary and secondary auditory fields as reflected by amplitude modulations in LFP recordings and changes in firing rate (Figure 4). Audiovisual interactions were detectable in both LFPs and spiking activity, and depended on stimulus efficacy as well as relative timing (Kayser et al., 2008). The addition of visual stimuli resulted in response modulations that ranged from enhancement (Figure 4, top LFP panel), to depression (Figure 4, LFP middle panel, all spiking examples), or no change (Figure 4, lower LFP panel). In a similar fashion, Ghazanfar and colleagues demonstrated multisensory integration of faces and voices using LFP and SUA analyses in the auditory cortex of rhesus monkeys (Figure 5) (Ghazanfar et al., 2005). Recent studies have begun to demonstrate that such subthreshold multisensory influences are more ubiquitous than previously realized and not restricted to cortical areas alone. For instance, traditionally the superficial layers of the SC were thought to be purely visual in nature and to play a role in visual form discrimination (Casagrande et al., 1972) but recent findings through LFP and multiunit activity (MUA) recordings demonstrate that visual activity is modulated by simultaneous presentation of an auditory stimulus (Ghose et al., 2012), which may in turn facilitate visual form discrimination under multisensory conditions. These studies illustrate the utility of LFP signal analysis in elucidating how modulatory influences from a second modality cue contribute to multisensory processing and how such modulatory influences might ultimately shape behaviors that rely on multisensory integration, such as form perception, vocalization, and communication.

**Figure 6:**
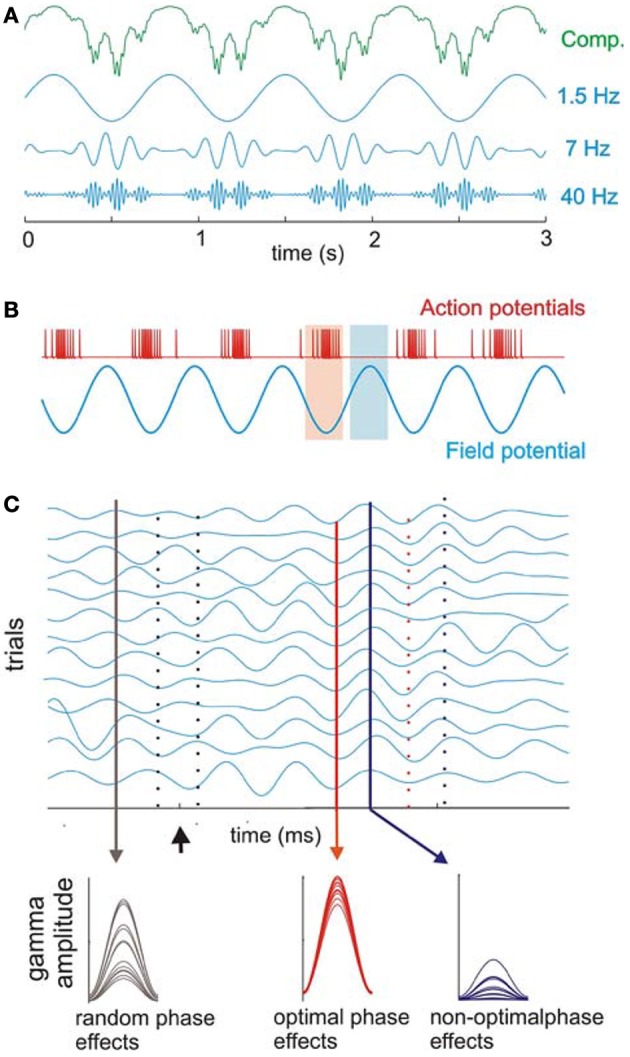
**Cross-frequency coupling between delta, theta, and gamma bands. (A)** Recorded oscillations (top, green) reflect complex (Comp.) combinations of components at different frequencies. Blue traces illustrate component delta (1.5 Hz), theta (7 Hz), and gamma (40 Hz) band oscillations that together make up the complex, combined signal (green illustration) recorded prior to filtering the signal into component frequency bands. Coupling between frequencies is hierarchical in organization such that gamma oscillatory amplitude varies with the phase of underlying theta oscillation, and theta in turn varies with the phase of underlying delta oscillation. **(B)** Action potential firing rate and local field potentials illustrate high and low excitability phases of ongoing neuronal oscillations. **(C)** Simulated single-trial responses demonstrate the effects of visual inputs on A1 activity. Oscillations within a given frequency are highly phase-variable across trials (black drop line on left) until a modulatory event (i.e., one that alters activity to a sub-threshold degree but does not reach significant enhancement or depression of the response; arrow) occurs. This event can cause a phase-reset of ongoing oscillations and can produce strong phase coherence. During such coherence, peaks, and troughs of ongoing oscillations align across trials (red solid and dotted lines, respectively) with both optimal and non-optimal phases that occur in temporally predictable patterns (red and blue lines illustrating low vs. high phase variability across trials, respectively). Sensory inputs (in this case, visual inputs to A1) can be timed such that they arrive either: (1) in random phase with the ongoing oscillation (black drop line in **C**) and generate highly variable response amplitudes (bottom; observed as random phase effects); (2) during the optimal phase (red drop line in **C**), amplifying the resulting signal (bottom, observed as optimal phase effects); or (3) during the non-optimal phase (blue line in **C**), suppressing the resulting signal (observed as non-optimal phase effects of the visual input across trials). Over time, the coherence observed across trials dissipates, and the system returns to resting (random phase) state [reprinted with permission from Schroeder and Lakatos (2009a), Figure 1. With kind permission from Springer Science + Business Media B.V.].

Beyond simple changes in LFP amplitude induced under multisensory circumstances, recent work has highlighted the utility of LFP and CSD analysis to yield insight into mechanistic questions within multisensory systems. For instance, Lakatos and colleagues used laminar CSD and multiunit activity analyses to demonstrate that in A1 of macaque monkeys the phase of ongoing oscillations is reset by somatosensory inputs (Lakatos et al., 2007). Such phase resetting of subsequent auditory inputs can be either enhanced or suppressed depending on the timing of the auditory and somatosensory stimuli relative to the oscillatory cycle. Each oscillation cycle of field potentials has periods of high and low excitability (Figures 6A,B, red and blue shaded areas, respectively) driving neurons toward or away from their firing threshold. During transient windows of opportunity, the phase of oscillation in the local neuronal ensemble can lock to relevant stimulus inputs (Figure 6C). Such phase-locking (i.e., synchronization) can serve to amplify neuronal representations, facilitate sensory discrimination, and increase response speed and accuracy (Lakatos et al., 2008; Schroeder and Lakatos, 2009b). Phase resetting to a high excitability state produces facilitation of responses to coincident sensory input, whereas phase resetting to a low excitability state yields suppression (Lakatos et al., 2007), allowing sharpened tuning of neuronal responses (O'Connell et al., 2011). Synchronization of cross-modal inputs likely underlie the enhanced discrimination, detection, and orientation behaviors observed behaviorally when multimodal stimuli are paired. This mechanism of crosstalk between sensory inputs is now providing an essential causal link between neuronal networks activity and behavioral gains. For instance, behaviorally, in the classic flash-beep test in which a visual (flash) and auditory (beep) are separated by an increasing degree of temporal offset (the stimulus onset asynchrony, or SOA), beyond a certain window of this offset (the “temporal binding window,” ~300 ms in normal human subjects), the stimuli are perceived separate rather than synchronous events (Shams et al., 2000; Powers et al., 2009; Foss-Feig et al., 2010). It seems likely that LFP recordings in the posterior superior temporal sulcus (pSTS) of non-human primates would reflect decreased phase coupling related to temporally offset audiovisual inputs (>300 ms), which would further manifest behaviorally as slower reaction times. However, synchronous flash-beeps would likely produce increased phase coupling, facilitation of responses to the coincident sensory inputs, and speeded reaction times demonstrating multisensory gains.

## Oscillations recorded through electroencephalography (EEG): population measures of fluctuations in neural activity

Beyond field potentials generated by local neuronal ensembles, larger neuronal populations demonstrate rhythmic shifts (oscillations) between states of high and low excitability on a more global scale (Buzsaki and Draguhn, 2004; Fries, 2005; Lakatos et al., 2005, 2008). These can be detected through EEG signals recorded on the scalp surface, or through the closely related blood oxygen level-dependent (BOLD) signal that forms the basis for functional magnetic resonance imaging (fMRI) (Logothetis et al., 2001; Viswanathan and Freeman, 2007; Rauch et al., 2008; Magri et al., 2012). Event-related potentials (ERPs) can be used to analyze EEG activity by averaging responses that are time-locked to stimulus presentation. In EEG recordings, higher frequency oscillations (i.e., gamma) are believed to be derived from coordinated activity in local neural assemblies whereas larger-scale networks are believed to be indexed through lower frequency oscillations (Von Stein and Sarnthein, 2000; Steriade, 2001; Csicsvari et al., 2003). These patterns create a dynamic hierarchy of neuronal oscillations with the flexibility to modulate both local and distributed network activity. A number of studies have associated the different frequency bands with distinct functional roles. Although by no means exhaustive, this includes linking *delta* (1–4 Hz) to motivational processes, reward, and deep sleep (Basar et al., 2000; Knyazev, 2007), *theta* (4–8 Hz) to working memory, emotional arousal, and fear conditioning (Jensen and Lisman, 2005; Knyazev, 2007), *alpha* (8–12 Hz) to working memory and awake resting state in the absence of sensory inputs (Palva and Palva, 2007), *beta* (12–30 Hz) to sensorimotor processing (Brovelli et al., 2004), and *gamma* (>30 Hz) to a variety of cortical functions including visual feature integration (Gray et al., 1989; Engel et al., 1991; Tallon-Baudry et al., 1996), attention (Muller et al., 2000; Fries et al., 2001), memory formation (Tallon-Baudry et al., 1998; Osipova et al., 2006), auditory processing (Kaiser et al., 2002; Debener et al., 2003), somatosensory processing (Bauer et al., 2006), olfactory processing (Wehr and Laurent, 1996), sensorimotor integration (Roelfsema et al., 1997), and movement preparation (Sanes and Donoghue, 1993). More recently, beyond simply looking at functional significance limited to certain oscillatory bands, studies have shown that phase synchrony and modulation across different frequency bands may be involved in complex tasks such as speech processing and memory encoding (Jensen and Lisman, 2005; Palva and Palva, 2007; Schroeder et al., 2008). Oscillatory activity also appears to be organized hierarchically, allowing sensory cortex to optimize the temporal structure of its activity pattern in order to best drive baseline excitability, and ultimately stimulus-driven responses (Schroeder et al., 1998; Lakatos et al., 2005; Schroeder and Lakatos, 2009a,b). In this way, fluctuations in the power and phase of oscillatory activity optimize the processing of rhythmic inputs and, through selective enhancement of neuronal response when sensory inputs arrive at an optimal phase of excitability for the neuronal ensemble, drive enhancement of perceptual and behavioral outcomes (Schroeder and Lakatos, 2009b).

## Oscillatory coherence as a tool in characterizing multisensory interactions

Although relatively understudied to date, oscillations have significant implications for multisensory processing, perception, and behavior. For instance, strength of synchronization was found to predict perception of ambiguous audiovisual stimuli as well as the integration of audiovisual information, particularly with respect to beta and gamma oscillations (Hipp et al., 2011). This finding implicates frequency-specific synchronization in widely distributed cortical networks as driving the formation of cross-modal associations. Other studies by Romei et al. demonstrated cross-modal phase locking of visual cortex activity (alpha oscillations) to the introduction of a sound. Phase-locking of the cross-modal stimuli introduced a periodicity that affected the pattern of phosphene perception, thus directly linking oscillatory phase-locking to behavioral outcomes (Romei et al., 2012). Thus, coherence of phase coupling between distinct brain areas may serve as a neural substrate influencing single-cell firing properties that ultimately bind anatomically segregated functional networks (Fries, 2005; Canolty et al., 2010). Specifically, phase coupling might subserve “integration through coherence,” thus bridging across different sensory modalities and enabling flexible, context-dependent binding that selectively strengthens those connections that are optimally adaptive for behavior (Engel et al., 1992, 2001; Singer, 1993; Gray, 1994; Singer and Gray, 1995; Salinas and Sejnowski, 2001; Fries, 2005; Womelsdorf et al., 2007; Senkowski et al., 2008; Benchenane et al., 2010). Given that phase-locked discharges of distributed neuronal assemblies are thought to be involved in binding stimulus features into a coherent percept (Gray et al., 1989; Engel et al., 2001), an essential aspect of multisensory processing, future experiments should target the effects of multisensory stimuli on phase coupling and coherence, particularly of beta and gamma frequency oscillations. For instance, targeting these frequency bands, changes in synchrony across subdivisions of the AES (the auditory subdivision, FAES; visual subdivision, AEV; and somatosensory subdivision, SIV) could be evaluated according to the degree of oscillatory congruence following presentation of auditory and visual motion cues. Using a saccade task to investigate accurate perception of motion direction and speed of reaction time, the prediction would be that synchrony would increase under audiovisual conditions in which auditory and visual motion occurred in the same direction. Increased synchrony would serve as a neural substrate for behavioral gains in directional assessment of congruent multisensory stimuli and would result in speeded, more accurate responses assessing the direction of stimulus movement. Furthermore, since the behavioral benefits of multisensory integration include enhanced detection and discrimination as well as speeded reaction times, it is noteworthy that stimulus discriminability is affected by whether task-relevant stimuli match an anticipated low-frequency rhythm of oscillatory activity (Jones et al., 2002; Praamstra et al., 2006; Lakatos et al., 2007, 2008; Schroeder et al., 2008; Schroeder and Lakatos, 2009a). Thus, the timing of sensory inputs from one modality relative to the phase of ongoing oscillations related to a second sensory modality is likely to be a key element in the multisensory enhancement or suppression of a response. Ultimately this would operate as an instrument of sensory selection in determining whether cross-modal stimuli are bound as a unified percept.

## Spike synchrony

It has been suggested that the activity of a group of neurons producing coincident spiking patterns forms an integral part of the neural code guiding behavior and perception (Shadlen and Newsome, 1994; Eggermont, 2001, 2006; Casagrande et al., 2002). These population-based approaches toward questions of neural encoding are becoming more commonly employed at all levels of the nervous system through analysis of correlated firing patterns, but their application toward understanding multisensory systems continues to lag behind studies within single sensory modalities.

Population encoding can be assessed in numerous ways. As highlighted in the earlier sections of this review, some of these indices include measures that are based largely on synaptic function, such as LFPs and oscillations. Others rely on spiking activity and include analyses of spike synchrony, the temporal correlation of spikes belonging to a group of neurons that are simultaneously recorded from either a local circuit or from distant areas (Singer, 1993; Singer and Gray, 1995; Usrey and Reid, 1999; Engel and Singer, 2001; Engel et al., 2001; Jermakowicz and Casagrande, 2007). Correlation of neural activity can refer to detection of temporal coincidences in the firing of two neighboring neurons, detection of co-variation in the firing rates of those neurons, or even co-variation in the postsynaptic activity generated by a cell's many inputs (Eggermont, 2007). This correlated neural activity can be measured using cross-correlograms (CCGs), an analysis tool that correlates activity between a pair of neurons and depict changes in the probability of a target neuron discharge relative to the discharge timing of a reference neuron. Time-locked discharges of a pair of neurons, known as coincident events, appear as peaks or valleys in the CCG and indicate excitatory or inhibitory interactions, respectively (Perkel et al., 1967; Gochin et al., 1989). A preferred method for such correlational analyses uses Joint Post-Stimulus Time Histograms (JPSTHs) due to their enhanced temporal resolution and facilitated observation of spike coincidence over time after a stimulus or behavioral event (Gerstein et al., 1989).

## Functional relevance of spike synchrony in sensory processing

Synchrony between single-unit pairs has been widely studied in visual cortical areas, and has revealed important features of correlated activity (for reviews see Singer, 1993; Engel et al., 1999; Usrey and Reid, 1999; Jermakowicz and Casagrande, 2007). Neurons with similar receptive field properties have synchronous discharges in the striate cortex of squirrel monkeys (Livingstone, 1996). Similarly, in extrastriate cortical areas such as the caudal STS of macaques, neurons exhibited synchronized activity that was dependent on specific stimulus properties (Kreiter and Singer, 1992; Gray and Viana Di Prisco, 1997). Spike synchrony in auditory cortex has been only minimally studied to date, though synchrony between auditory cortical neurons has been implicated in processing sound movement and localization (Ahissar et al., 1992). As described in visual areas, the correlation strength of auditory cortical neurons appears to depend on the receptive field properties of paired neurons and has been specifically associated with spectro-temporal receptive fields, binaural interactions, and temporal response properties (e.g., response onset/offset as well as the temporal pattern of discharge) (Brosch and Schreiner, 1999; Eggermont, 2006).

**Figure 7:**
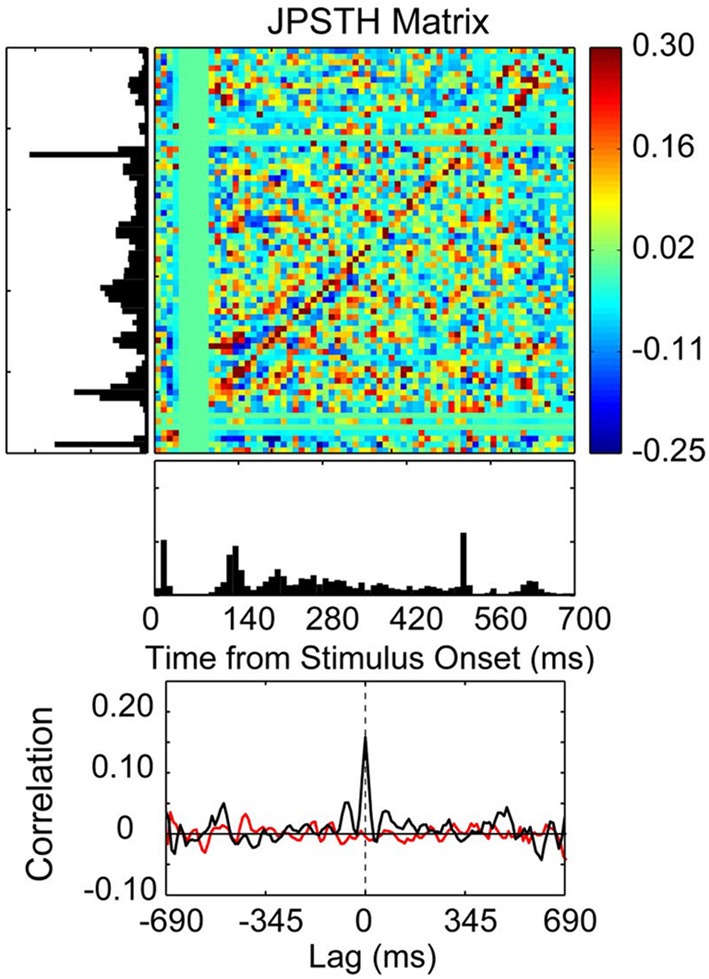
**Correlated spike activity recorded from adjacent electrodes in a 10 × 10 multielectrode array recording from the thenar pad representation of area 3b in an owl monkey**. Spike synchrony between two neurons is shown in a JPSTH matrix, with a psth for each neuron shown to the left and below to illustrate responses to skin indentations of the thenar pad and digital (P1) pad for 100 trials. The colored pixels in the JPSTH matrix represent the magnitude of the normalized correlation at different lag times over a poststimulus time of 700 ms. The magnitude of the normalized correlation shown in the JPSTH and in the cross-correlation histogram reveal a strong spike synchrony that occurred at 0 ms lag time. The cross-correlation histogram (black) revealed a peak correlation of 0.16 that exceeded the mean correlation from the shuffled trials (red). The time-averaged cross-correlogram was computed by summing the JPSTH bins parallel to the main diagonal, measuring the average positive or negative correlation across the entire interval of analysis in 1 ms bins [reprinted with permission from Reed et al. (2008)].

Neural synchrony has also been demonstrated in somatosensory processing such that the degree of correlated activity parallels the extent of receptive field overlap for neurons in primary and secondary somatosensory areas (Dinse et al., 1993; Roy and Alloway, 1999; Alloway et al., 2002). Neural synchronization may also encode more complex stimulus features such as movement (Roy and Alloway, 1999), discrimination, and localization (Figure 7) (Reed et al., 2008) as well as surface texture (Wolfe et al., 2008; Jadhav et al., 2009). Spike synchrony may also be attentionally modulated (Roy et al., 2000; Steinmetz et al., 2000), serving to increase the signal-to-noise ratio for the stimulus-driven response (Crick and Koch, 1990; De Oliveira et al., 1997; Salinas and Romo, 2000; Fries et al., 2001) with behavioral outcomes such as improving performance during a vibrotactile discrimination task (Romo et al., 2003). However, it is also important to examine the noise correlation of simultaneously recorded neuronal pairs, which can affect the information in population coding of responses and ultimately influence computational strategies in neuronal networks (Averbeck et al., 2006). Through such noise correlations, small changes in individual neuron activity can have a large impact at the population scale, altering both the encoding and decoding of a signal. Ultimately, analyses of spike synchrony may provide insights into not only the temporal aspects of neural coding but also their coordinating role in sensory perception, potentiating the activity of targeted neural networks and bridging between sensory modalities to generate a unified percept.

## Spike synchrony: applications for understanding multisensory interactions

Although spike synchrony has been widely studied within individual sensory systems, surprisingly few studies have examined coincident firing patterns and their role in multisensory encoding (e.g., Ghoshal et al., 2011). Future studies of particular interest would utilize awake animal recordings to target multisensory neurons in cortical areas such as the AES, the ventral intraparietal area (VIP), and the STS in order to examine how spike synchrony contributes to behavioral gains and perceptual benefits conferred by cross-modal stimuli. It can be postulated that spike synchrony might be linked to integrative processing and its behavioral manifestations through stimulus feature encoding, attentional modulation, and increasing the signal-to-noise ratio of correlated activity in multisensory neurons. This could be examined through an experiment in which animals are trained to perform a visual detection task while a multielectrode array records neuronal activity spanning distinct regions of visual cortex. Behavioral performance and neuronal activity would then be compared under visual vs. audiovisual conditions. One underlying hypothesis of these experiments would be that multisensory integration might not manifest itself through changes in firing rate, but rather through increased spike synchrony between neuronal pairs (either within the same visual area, between visual areas, or a combination of both). Furthermore, increased spike synchrony might be correlated with behavioral manifestations such as enhanced stimulus detection (e.g., greater response accuracy, speeded reaction time, enhanced detection or discrimination) under multisensory conditions.

## Signal detection theory: receiver operating characteristic (ROC) analysis and implications for multisensory processing

The nervous system is faced with the incredible challenge of successfully extracting valuable information from a highly noisy environment. The sources of noise can be manifold and broadly belong to two categories: (1) external noise, such as that generated within the environment, and (2) internal noise, such as that generated within the nervous system. Signal detection theory (see Green and Swets, 1966; Heeger, 1997, 2003; Macmillan and Creelman, 2004, for review) is a conceptual framework that has great utility in extracting meaningful signals in the presence of noise, and in relating neural activity to behavioral outcomes. Responses in a signal detection framework are generally divided into a 2 × 2 array, comprised of: (1) a hit (successful detection of signal), (2) a false alarm (detection of a signal when there is none), (3) a miss (failure to detect a signal when it is present), or (4) a correct rejection (detection of no signal when there is none). When quantifying responses, the criterion threshold level (also called response bias, or decision bias, and expressed as β) greatly affects the outcome of a signal detection task.

Discrimination of a signal from noise depends primarily on the *separation* of the noise from the combined signal and noise distributions, as well as the spread of the two distributions or the amount of overlap. Higher noise levels involve more overlap and hence greater spread whereas lower noise levels involving reduced overlap and spread, enabling easier signal detection. This can be expressed by the discriminability—or sensitivity—index (d′):

$${\text{d}}^{\prime }=\text{separation}/\text{spread}$$

where separation is the difference between the means of the two distributions (noise and signal), spread is the standard deviation of the distributions, and d' is a true measure of the internal response free from subjective bias. All possible outcomes of a signal detection task can be expressed in a single curve—the Receiver Operating Characteristic (ROC) curve—that is dependent on the criterion chosen. ROC curves are generally expressed with false alarm rate on the x-axis and hit rate on the y-axis. When a signal is detected reliably, the area under the ROC curve is higher, but higher false alarm rates (unreliably detected signals) result in decreased area under the curve.

**Figure 8:**
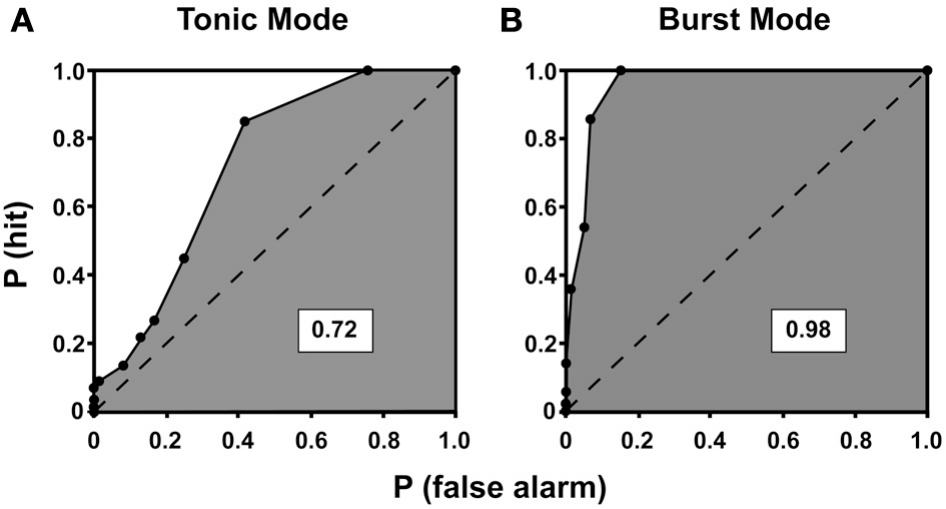
**ROC curves for tonic (A) and burst (B) mode of response for a neuron recorded from the lateral geniculate nucleus of a cat**. The ROC curves plot the probability of correctly detecting visually-driven activity from spontaneous activity. The probability of detecting the signal, P(hit), is plotted on the y-axis against the probability of mistaking spontaneous activity for visually driven activity, P(false alarm), at all possible criterion levels. The area under each curve is shaded, and the dashed line divides the total area of the curve in half. A value of 1 indicates perfect detection of signal from noise whereas a value of 0.5 indicates that signal and noise are indistinguishable. Here, the area under the curve is higher for burst mode compared to tonic mode, indicating enhanced signal detection conferred by the burst mode of response [reprinted with the permission of Cambridge University Press and adapted from Guido et al. (1995)].

Numerous studies performed in the visual system have used analyses derived from signal detection theory to test the ability of neuronal responses to predict stimulus characteristics and/or behavior (Tolhurst et al., 1983; Bradley et al., 1987; Britten et al., 1992; Guido et al., 1995; Thompson et al., 1996). However, because single neuron responses in isolation provide little information about stimulus characteristics, the responses of multiple neurons tuned to different but overlapping ranges of stimulus dimensions must be considered when predicting psychophysical discrimination thresholds. Otherwise, it is possible for some neuronal responses to reflect smaller differences in stimulus features (such as the orientation of a bar of light) than those observed through behavioral measures (e.g., discrimination thresholds). Using the slope of ROC curves, Bradley et al. showed that single neurons in the visual cortex can reliably signal stimulus orientation and spatial frequency differences that were considerably smaller than their tuning widths (Bradley et al., 1987). Similarly, studies recording from the FEF have demonstrated that at the single neuron level there exists an explicit dissociation between perceptual processing and response generation (Thompson et al., 1996). By plotting the area under the ROC curves as a function of time for a simple popout visual search discrimination task, it was found that the activity of FEF neurons could reliably discriminate targets from distractors after 130 ms of search stimulus array presentation. In addition, a separate study showed that presaccadic movement neurons in FEF are activated ~100 ms before saccades and that only when their activity reaches a certain threshold does a saccade occur (Hanes et al., 1995; Hanes and Schall, 1996). These studies illustrate the utility of ROC analyses in differentiating between two stages of processing in the visual system—target discrimination and response generation. Furthermore, studies in the LGN have elucidated how distinct neuronal response modes (burst vs. tonic) relate to signal detection ability in visual processing (with higher signal detection characterizing the burst mode; Figure 8) (Guido et al., 1995). Thus, ROC analyses can be used to reveal the dynamic relationship between various aspects of stimulus processing, neuronal activity, and ultimately behavior.

Neuronal recordings in awake animals performing a multisensory task, in conjunction with ROC analyses, would provide the necessary link between neuronal responses and behavioral outcomes that has been critically absent in the multisensory field. The behavioral benefits revealed by psychometric functions (such as greater response accuracy and speeded reaction times) seen for cross-modal trials would be predicted to coincide with increased ROC areas computed from neuronal recordings as the animal performed a task (e.g., a saccade in the direction of perceived audiovisual motion). Additionally, the time course of the neuronal response plotted as a function of ROC area would help to distinguish the contribution of neuronal activity to different stages of the task being performed, thereby directly relating multisensory neural activity to behaviorally and perceptually relevant outcomes. Recent studies have begun to study such neurometric/psychometric relationships using ROC analysis in a multisensory context. For instance, simultaneous recording of neuronal activity in the dorsal medial superior temporal (MSTd) area of monkeys was performed during a multisensory discrimination task using visual and vestibular cues to discriminate heading (Gu et al., 2008). Results of this study revealed that MSTd neuronal activity closely paralleled behavioral choice with respect to improvements in both perceptual sensitivity and cue weighting, demonstrating direct neural links to multisensory enhancement of behavior.

## Summary: convergent approaches toward understanding sensory integration

An increasing number of studies have begun to address interactions across multiple sensory modalities, improving our understanding of the neurophysiological mechanisms that underlie behavioral and perceptual outcomes of cross-modal processing. Going forward, methodologies that have advanced our understanding of individual sensory systems in isolation can be applied toward bridging the gap of how these senses interact to form a unified percept of our surroundings. Analyses that go beyond classical firing rate measures to assess multisensory gain within the realms of response variability, LFPs, CSD, oscillatory coherence, spike synchrony, and ROC are promising tools for understanding the neural encoding of multisensory stimulus features. Analytical tools including temporal coding, response variability (FF), and measures of signal detection outcomes (ROC analyses) are integral in relating the reliability and efficacy of neural processing to behavioral gains such as improved target detection under multisensory conditions. Sensory integration, in part, also requires binding of unisensory representations through interactions between sensory cortices. This in turn requires widely distributed functional coupling, poising slow frequency bands of LFP or CSD signals as a promising experimental focus, given that these bands have been particularly implicated in long-range interactions. Future studies should address direct links between oscillatory activity, perception and behavior as it relates to cross-modal processing. Furthermore, using multisite and multiarea recordings in behaving animals to examine cross-talk between neurons of different sensory areas through increased or decreased spike synchrony, independent of changes in firing rate, might elucidate how cue weighting of significant sensory events works to perceptually bind stimuli as a unified percept or dissociate them (Fetsch et al., 2013). Of course the current review is by no means comprehensive, and other methods constitute critical tools for encapsulating multisensory processing, including mutual information theory, discriminant analysis, Bayesian modeling, and population vectors. Ultimately, mechanisms such as phase coupling and oscillatory synchronization, modulations in LFPs or CSD, and changes in response variability, signal detection, and spike synchrony are likely to work in concert to varying degrees in order to effectively integrate sensory cues across modalities, optimize behavior, and ultimately derive perceptual meaning from our sensory surroundings as a synthesized whole.

## Funding

This work was supported by National Institute of Mental Health Grant MH-63861 and Vanderbilt Kennedy Center Development Funds to Mark T. Wallace.

### Conflict of interest statement

The authors declare that the research was conducted in the absence of any commercial or financial relationships that could be construed as a potential conflict of interest.
